# Comparison of Manual and Automated Nucleic Acid (RNA) Extraction Methods for the Detection of SARS-CoV-2 by qRT-PCR

**DOI:** 10.7759/cureus.36773

**Published:** 2023-03-27

**Authors:** M Dhibika, N S Madhusudhan, A Malini, Mailan Natarajan

**Affiliations:** 1 Microbiology, Indira Gandhi Medical College & Research Institute, Puducherry, IND

**Keywords:** sars-cov-2, rna extraction, qrt-pcr, covid-19, automation

## Abstract

Objectives

During the COVID-19 pandemic, several laboratories used different RNA extraction methods based on the resources available. Hence this study was done to compare the Ct values in qRT-PCR, time taken (sample processing-loading to PCR), manpower requirement, and cost of consumables between manual and automated methods.

Materials and methods

A cross-sectional study was done on 120 nasopharyngeal/oropharyngeal swabs received in VRDL for RT-PCR testing. Based on the results of automated RNA extraction (Genetix, HT 96 Purifier) and RT-PCR (Trivitron PCR Kit) detecting E gene (screening) and ORF gene (confirmatory), the division into Group- I (Ct 15-22), Group- II (Ct 23-29), Group-III (Ct 30-36) and Group-IV (Ct >36) was done. Manual RNA extraction was done using magnetic beads (Lab system, Trivitron).

Statistical analysis

Data were analyzed by SPSS 19.0 version software. Ct values obtained in the two methods were compared by paired t-test, GroupWise. Z test was used to compare the other parameters.

Results

The difference in Ct values for target genes was statistically significant (p<0.05) in Group-I to III; however, no variation in result interpretation. The difference in time, manpower, and cost were statistically significant (p<0.05). The manual method required twice more manpower; 40 minutes more time & automated method cost 3.5 times more for consumables.

Conclusion

The study showed that RNA yield was better with automated extraction in comparison to manual extraction. The samples extracted by the automated method detected the virus at a lower Ct range by PCR than the manual method. Automated method processed samples in less time and with less manpower. Considering the cost factor, manual extraction can be preferred in resource-limited settings as there was no difference in the results of the test. The manual method requires more hands-on time with potential chances of cross-contamination and technical errors.

## Introduction

The coronavirus disease 2019 (COVID-19) is a novel disease caused by a highly contagious virus called severe acute respiratory syndrome coronavirus 2 (SARS-CoV-2). The emergence of this novel virus was first observed in Wuhan city, China. Later it was declared a global pandemic by the World Health Organization (WHO) in March 2020 [1]. Global reports of more than 600 million confirmed cases with a mortality of 6 million were documented by WHO. In India, 44 million cases were confirmed with 5 lakh deaths till date by WHO [2].

SARS-CoV-2 is one of the seven human-infecting coronaviruses made up of single-stranded positive sense enveloped RNA virus (+) ssRNA belonging to the P-coronavirus lineage B with a genome of 27-32 kb encoding four structural proteins (S, E, M, N) and 16 non-structural proteins namely nsp 1-16 [2,3]. Major transmission routes are droplet, aerosol, and contact transmission thereby posing a higher transmission rate. Clinical presentation varies ranging from mild respiratory illness to severe manifestations like acute respiratory distress syndrome [4]. Laboratory testing aids in early diagnosis and thereby plays an essential role in containing the spread of this pandemic [5]. Real-time reverse transcription-polymerase chain reaction (qRT-PCR) is recommended by the WHO for accurate detection and quantification of SARS-CoV-2 [5,6].

PCR accuracy and its analytical performance are largely dependent on the quality of samples and nucleic acid (RNA) extraction efficiency [7]. The genomic RNA of SARS-CoV-2 can be extracted from nasopharyngeal/oropharyngeal swabs either by using manual or automated methods [8]. Manual extraction methods are arduous, labor-intensive, and require well-trained personnel, and batch-to-batch inconsistency has been observed [9,10].

Several extraction methods are being used during this pandemic due to high demand and shortage in the supply of kits [11].^ ^The analytical performance of both methods is very limited and further evaluation is still essential with special reference to clinical specimens prone to harbor PCR inhibitors. Hence this study was carried out to elucidate the comparative performance of manual and automated RNA extraction methods for the detection of the SARS-CoV-2 virus using qRT-PCR.

This article was previously presented as an oral paper in the National E-Conference on Clinical Microbiology and Infectious Diseases 2021 organized by Madras Medical College & RGGGH Chennai on December 4, 2021.

## Materials and methods

It was a prospective cross-sectional study conducted in Virus Research and Diagnostic Laboratory (VRDL) of our institute, during August and September 2021 after obtaining ethical clearance from Institute Ethical Committee (Human studies) with IEC approval (No.342/IEC-32/IGMC&RI/PP-22/2022).

Inclusion and exclusion criteria

Nasopharyngeal/oropharyngeal swabs submitted in VRDL as per ICMR guidelines for RT-PCR testing were included in the study. Samples that were tested by automated method and had passed the quality control were selected for extraction by manual method. Purposive sampling was done based on the Ct values results obtained for the ORF1ab gene in automation method (HT 96 Purifier, Genetix, India) and they were divided into four subgroups namely Group- I (Ct 15-22), Group- II (Ct 23-29), Group-III (Ct 30-36) and Group-IV (Ct >36) having 30 samples in each group.

These samples were processed in accordance with national biological safety regulations and RNA was extracted as per manufacture instructions and tested by qRT-PCR (COVIDsure, Trivitron, India) detecting the viral targets - E gene as screening and ORF1ab as confirmatory gene and RPP-30 as the internal control. These nasopharyngeal/oropharyngeal samples were stored in the Eppendorf tubes at -80°C for manual RNA extraction.

Automated RNA extraction

A magnetic bead-based kit (HT 96 Purifier, Genetix, India) was used for automated RNA extraction. The extraction procedure involves four steps: sample lysis, nucleic acid (RNA) binding to magnetic beads covered with silicon dioxide, washing, and elution. All steps were conducted according to the manufacture instructions in 96 deep well plates for approximately 35 mins. Ct values were recorded for all three genes.

Manual RNA extraction

Manual extraction was done in a Biosafety cabinet using a magnetic beads kit (Lab System, Trivitron). The samples were extracted based on the manufacture recommendations. Virus-inactivated samples stored in the Eppendorf tubes at -80°C were thawed and 40 µL of magnetic beads were added to the sample, vortexed, and placed in the magnetic stand. This is followed by the addition of 200 µL of isopropanol to each sample and the samples were thoroughly vortexed. The samples were washed twice using wash buffer ethanol and the flow through was discarded. Lastly, the columns were air dried for 5 minutes at 55°C in the incubator and the RNA samples were eluted using 50 µL elution buffer. Elute of volume 5 µL is added to the master mix and PCR was run.

Specific target detection by qRT-PCR

RNA extracted through both manual and automated extraction methods were amplified in the CFX 96 thermal cycler (Bio-Rad, India) using a kit, (RNAsure, Trivitron, India) targeting primers for E gene of Sarbecovirus in HEX channel, ORF1ab gene of SARS-CoV-2 in FAM channel and human housekeeping gene RPP-30 as the internal control in ROX channel.

Data analysis

Data were analyzed by SPSS 19.0 version software. Ct values of manual and automated methods were compared by Paired t-test, GroupWise. Time taken from sample processing to loading to PCR, cost comparison, and manpower requirement between manual and automated extraction methods was compared by Z test. The difference in Ct values between the manual and automated methods was analyzed in terms of mean and standard deviation. Comparisons were considered statistically significant if p-value<0.05.

## Results

The Ct values obtained in automated and manual methods in Group I-IV are shown in Table 1. The manual method yielded slightly higher Ct value ranges in comparison with automated RNA extraction. The difference in Ct value between the two methods was statistically significant (p-value<0.05) in groups I-III, which did not influence the final result interpretation.

**Table 1:** Comparison of Ct values between automated and manual methods

Gene	Automation Ct values (Mean + Standard deviation)	Manual Ct values (Mean + Standard deviation)	P-value (Paired t-test)
GROUP-I (HIGH POSITIVE) (n=30)			
ORF	19.9±2.1	21.5±4.9	0.071
E GENE	21.1±2.2	22.8±4.2	0.019
RPP30	30.4±1.9	29.9±10.4	0.772
GROUP-II (MEDIUM POSITIVE) (n=30)			
ORF	25.7±2.1	28.2±2.2	0.000
E GENE	27.2±2.2	28±2.3	0.005
RPP30	29.5±6.2	31.8±2.5	0.068
GROUP-III (LOW POSITIVE) (n=30)			
ORF	33±1.7	28.01±11.7	0.034
E GENE	31.3±8.7	31.6±9.1	0.898
RPP30	29.9±5.9	29.8±1.4	0.944
GROUP-IV (NEGATIVE) (n=30)			
ORF	NA (Not amplified)	NA	-
E GENE	NA	NA	-
RPP30	33±2.2	35±2.5	-

A comparison of time taken, manpower requirement and cost of consumables in the two methods is shown in Table 2. The difference in all three parameters in the two methods was statistically significant.

**Table 2:** Comparison of cost of consumables, time, and manpower requirement between automated and manual method

Parameters	Manual method (Mean+Standard deviation)	Automated method (Mean+Standard deviation)	p-value (Z test)
Time(minutes)	149.8±29.8	110.7± 7.7	0.000
Manpower	6.4±0.8	3±0.4	0.000
Cost(rupees)	5243.85±105.02	18138.64±363.20	0.000

## Discussion

We evaluated the comparative performance of two RNA extraction techniques for SARS-CoV-2 detection by qRT-PCR with the aim to check whether these techniques can be inter-changeable in crises when supply chains are limited and unreliable. Rapid and sensitive diagnostic modality is necessary to contain and control this pandemic. Nucleic acid (RNA) detection by RT-PCR is still the gold standard test to diagnose COVID-19 infection [11,12]. The quality and quantity of the extracted nucleic acid (RNA) significantly influence the results. Thus, the method used for RNA extraction is the most important variable to determine the positivity of the sample for the SARS-CoV-2 genome, especially for those labs that are not equipped with automated extraction systems [13]. In resource-limited settings, using manual RNA extraction methods for COVID-19 diagnosis should choose ICMR-approved kits for reliability [14]. Though the automated method is capable of processing more samples in a much shorter time, the cost is relatively higher than manual extraction. A study has also shown that the RNA yield using manual extraction shows higher variation compared to automated, which proves that automated extraction is more standardized and consistent [15].

A statistically significant difference in Ct values of target genes was found between the two methods in our study similar to studies done by Karoline et al. [13] and Ransom et al. [16]; however, it did not affect the result interpretation. A study done by Kumar et al. [17] compared two automated platforms with manual RNA extraction and found equally good efficacy in RNA extraction between manual and one of the automated methods.

A similar study observed no significant difference in the RT-PCR positivity rate between the two methods statistically [18]. Another study done in Brazil observed 100% sensitivity in automated extraction when compared to manual and rapid extraction methods [13]. A study by Zouré et al. [19] found no statistically significant difference in the mean Ct value of the ORF1ab gene between the automated and manual RNA extraction from frozen samples which is in contrast to our study. A study done by Bhargav et al. [20] observed significantly higher test failure in the manual extraction than in the automated method. A review article on using a modified DNA extraction kit found that RNA extraction efficiency was better in automated method [21] which is similar to our observation. The cost analysis done by Karoline et al. [13] showed that the manual method is less expensive, similar to our study.

However, it is essential to consider that Ct values might be affected by pre-analytic, analytic, and post-analytical variables such as collection technique, specimen type, sampling time, viral kinetics, transport and storage conditions, nucleic acid extraction (RNA), viral RNA load, primer designing, real-time PCR efficiency [22,23]. Result interpretation as positive/negative was not influenced by the method of extraction though there was a difference in Ct value between the methods.

## Conclusions

The study showed that RNA yield was better with automated extraction in comparison to manual extraction. The samples extracted by the automated method detected the virus at a lower Ct range by PCR than the manual method. Automated method processed samples in less time and with less manpower. Considering the cost factor, manual extraction can be preferred in resource-limited settings as there was no difference in the results of the test. The manual method requires more hands-on time with potential chances of cross-contamination and technical errors.
